# Pumpkin powdery mildew disease severity influences the fungal diversity of the phyllosphere

**DOI:** 10.7717/peerj.4559

**Published:** 2018-04-02

**Authors:** Zhuo Zhang, Luyun Luo, Xinqiu Tan, Xiao Kong, Jianguo Yang, Duanhua Wang, Deyong Zhang, Decai Jin, Yong Liu

**Affiliations:** 1 Hunan Academy of Agricultural Sciences, Hunan Plant Protection Institute, Changsha, Hunan, China; 2 College of Bioscience & Biotechnology, Hunan Agricultural University, Changsha, Hunan, China; 3 Chinese Academy of Sciences Key Laboratory of Environmental Biotechnology, Research Center for Eco-Environmental Sciences, Chinese Academy of Sciences, Beijing, China; 4 Vegetable Research Institute, Hunan Academy of Agricultural Science, Changsha, Hunan, China

**Keywords:** Disease severity, Powdery mildew, Illumina MiSeq, Phyllosphere microbiota, Fungal community

## Abstract

Phyllosphere microbiota play a crucial role in plant-environment interactions and their microbial community and function are influenced by biotic and abiotic factors. However, there is little research on how pathogens affect the microbial community of phyllosphere fungi. In this study, we collected 16 pumpkin (*Cucurbita moschata*) leaf samples which exhibited powdery mildew disease, with a severity ranging from L1 (least severe) to L4 (most severe). The fungal community structure and diversity was examined by Illumina MiSeq sequencing of the internal transcribed spacer (ITS) region of ribosomal RNA genes. The results showed that the fungal communities were dominated by members of the Basidiomycota and Ascomycota. The *Podosphaera* was the most dominant genus on these infected leaves, which was the key pathogen responsible for the pumpkin powdery mildew. The abundance of Ascomycota and *Podosphaera* increased as disease severity increased from L1 to L4, and was significantly higher at disease severity L4 (*P* < 0.05). The richness and diversity of the fungal community increased from L1 to L2, and then declined from L2 to L4, likely due to the biotic pressure (i.e., symbiotic and competitive stresses among microbial species) at disease severity L4. Our results could give new perspectives on the changes of the leaf microbiome at different pumpkin powdery mildew disease severity.

## Introduction

Powdery mildew is a common fungal disease of cucurbits and the major cause of losses in cucurbit production worldwide (Bellon-Gómez et al., 2011). *Golovinomyces cichoracearum* (syn. *Erysiphe cichoracearum*) and *Podosphaera xanthii* (syn. *Sphaerotheca fuliginea*) are the two main pathogenic fungi that cause powdery mildew in the cucurbits (Lebeda, Mggrath & Sedlakova, 2010). Impacts of powdery mildew on crop production include reduced photosynthesis, impaired growth, premature senescence, and yield loss (Lebeda, Mggrath & Sedlakova, 2010). The powdery mildew pathogen lives with the obligate biotrophic lifestyle (Hacquard, 2014). Powdery mildew symptoms first appear as pale, chlorotic spots on leaves that soon turn powdery-white in appearance (fungal spores) and start on the crown and lower leaves, mainly on the under-leaf shaded surface (Lebeda, Mggrath & Sedlakova, 2010). Young plants may turn yellow, stunted, and may die, and then severely infected leaves become brown and brittle, resulting in foliage loss (Lebeda, Mggrath & Sedlakova, 2010).

The phyllosphere or leaf surface is an important microbial habitat for members of the major bacterial and fungal groups, and Archaea (Lindow & Leveau, 2002; Lindow & Brandl, 2003). These microorganisms play a crucial role in helping their host against pathogens (Lacava et al., 2006; Mejía et al., 2008; Rajendran et al., 2011). In past years, many studies focused on screening plant growth-promoting microorganisms which can help us manage plant diseases (Compant et al., 2005; Everett et al., 2005; Hirano & Upper, 2000; Whipps et al., 2008). However, not all the microbes in the natural environment are considered culturable (Kimura, 2006). In the past few years, the development of next-generation rRNA sequencing techniques has enabled us to obtain in-depth descriptions of the composition of the microbial communities associated with leaves of *Arabidopsis thaliana* (Reisberg et al., 2013), potatoes (Becker et al., 2008), rice (Mwajita et al., 2012), spinach (Lopez-Velasco et al., 2011; Lopez-Velasco et al., 2013), grape tree (Leveau & Tech, 2011), and various tree species including salt cedar (Redford et al., 2010; Finkel et al., 2011).

Historically, scholars began to study the rhizosphere as a microbial habitat as early as 100 years ago (Hartmann, Rothballer & Schmid, 2008) and the importance of microbial communities is well recognized in plant health and growth. Although the root–rhizosphere microbiome has been well-studied now, much remains to be understood about the plant-microbe interactions occurring in the phyllosphere. Now, the development of new high-throughput sequencing technologies enables researchers to better understand the microbiome fields, especially the phyllosphere microbiome. Not only can it help us understand the communities better, but it can also help us study the interactions between host plants and the environment deeply.

As sessile organisms, plants are affected by environmental stresses during their growth period (Zhang et al., 2014). The phyllosphere microorganisms are influenced by both biotic and abiotic factors, some of which are fairly stable and constant, such as habitat conditions (Yang et al., 2016; Fonsecagarcía et al., 2016), the host genotype (Sapkota et al., 2015; Bodenhausen et al., 2014; Hunter, Pink & Bending, 2015), elevation gradient (Cordier et al., 2012; Zhang et al., 2015), and seasonal variation (Copeland et al., 2015; Jackson & Denney, 2011; Davey et al., 2012). Microbial interactions in the phyllosphere play an important role in the agroecosystem, not only affecting the health and growth of plants in natural communities, but also the productivity of agricultural crops (Whipps et al., 2008). The phyllosphere is constituted of a high proportion of plant-beneficial microorganisms such as antagonists, diazotrophs, and plant growth-promoting bacteria (PGPB) that colonized plant-associated habitats, but also plant pathogens and potential human pathogens (Berg, Eberl & Hartmann, 2005). Plants can protect themselves against pathogenic fungal infection by natural means which include biological and non-biological inducers (Shi et al., 2007). However, less is known about the colonization and persistence of nonpathogenic microbes on this extensive habitat, as well as about their interactions with pathogenic microorganisms, and impact of single strains in the microbial community. The rhizosphere community of specific biocontrol agents have shown minor and only transient effects according to the risk assessment and colonization studies (Scherwinski, Wolf & Berg, 2007; Adesina et al., 2009; Chowdhury et al., 2013; Schmidt et al., 2012), while impacts of pathogens on the phyllosphere microbiome are largely underexplored. To the best of our knowledge, although there are fewer studies about the relationship between the phyllosphere microbiome and pathogen using Illumina sequencing technology, the existing results still showed that microbes present on the plant surface play an important role in the resistance to the pathogen (Ritpitakphong et al., 2016; Vogel et al., 2016; Busby, Peay & Newcombe, 2016).

In this study, we intended to (1) further explore the interaction between the pathogen *Podosphaera* and other dominant microorganisms and (2) gain a better understanding of the theoretical basis for disease control in agroecological systems by evaluating whether the diversity and community structure of pumpkin (*Cucurbita moschata* Duchesne ex Poir) phyllosphere microbiota is influenced by the abundance of the pumpkin powdery mildew pathogen *Podosphaera*. We analyzed the fungal communities of 16 pumpkin leaf samples showing symptoms of powdery mildew disease with different disease severity levels ranging from L1 (least severe) to L4 (most severe) by sequencing the ITS regions of fungal rRNA genes using Illumina MiSeq. The richness and diversity of the fungal community were compared across disease severity levels, and statistical analyses based on OTUs or taxonomic classification were also performed. These results are indicative for new perspectives on the changes of leaf microbiome at different pumpkin powdery mildew disease severity.

## Materials and Methods

### Site and sampling

Leaf samples were randomly collected from pumpkin (*C. moschata*:*nen zao 1*) plants showing symptoms of powdery mildew disease. The samples were collected in June 2015 in the base of Vegetable Research Institute, Changsha, Hunan Province, China. The field was divided into four adjacent experiment areas, planted with the same type of pumpkin NZ number 1. The leaf samples were divided into four groups (L1–L4) based on the proportion of lesion area every leaf; L1 (no lesions), 6%<L2<11%, 11%≤L3<20%, L4≥40%, respectively. The classification method of disease level was applied based on the National standard of China (GB/T 17980.30-2000). According to four different disease severities (disease grade: 1–4), the same sizes of 10 pumpkin leaves (same disease severity, different individual) were randomly collected and mixed into sterile bags in each area, and all the leaves were from different pumpkin plants at fruiting stage. Each area was sampled using five-point sampling within an area of 30 m^2^. Leaf samples were collected in separate bags at refrigerated temperature, and were transferred to the laboratory for processing. Each of the 10 leaves in each bag were cut into tiny pieces and mixed. To harvest microbes on the leaf surface, 10 g of leaf were submerged in 100 mL of PBS with 0.01% Tween-80 in a 250 mL sterile conical flask. The flask was shaken at 250 rpm for 30 min at 28 °C, and then subjected to ultrasound for 10 min. The microbes were then harvested using air pump filtration using a 0.22 µm filter. The microfiltration membrane was stored at −20 °C until used.

### DNA extraction and purification

The MP FastDNA^®^ SPIN Kit for soil (MP Biochemicals, Solon, OH, USA) was used to extract DNA from the leaf surface samples according to the manufacturer’s protocol. DNA was extracted from the microbes harvested from the leaf surface. PCR amplicon libraries were prepared for each sample (DNA concentration at 30 ng/µL) using the eukaryotic primers ITS5 (5′-GGAAGTAAAAGTCGTAACAAGG-3′) and ITS2 (5′-GCTGCGTTCTTCATCGATGC-3′) with the forward primer modified to contain a unique 6 nt barcode at the 5′ end. Fungal ITS1 regions were amplified in a total volume of 50 µL that contained 1 µL (5 µM) of each forward and reverse primer, 1.5 µL of dNTP mix (30 mM each), 0.5 µL of 5 U *Taq* DNA polymerase (TaKaRa), 5 mL of 10 × PCR buffer (with Mg^2+^) and 1 µL of DNA. Reaction conditions consisted of an initial denaturation step at 94 °C for 1 min, followed by 35 cycles of denaturation at 94 °C for 20 s, primer annealing at 57 °C for 25 s, and extension at 68 °C for 45 s, and then a final extension at 68 °C for 10 min. PCR products with a bright band of between 250 and 450 bp were collected by agarose gel electrophoresis and purified with an E.Z.N.A.^®^ Gel Extraction Kit. The purified PCR amplicons were pooled in equimolar amounts using Qubit (CA, USA) and paired-end sequenced (2 × 250 bp) on an Illumina MiSeq platform by ANNOROAD Gene Technology Co., Ltd. (Beijing, China) according to standard protocols.

### Processing of sequence data

After the MiSeq sequencing, the raw sequence data reads in fastq format were collected. Separate files were generated based on the forward and reverse directions and the barcodes. Paired end reads were merged using the FLASH program (Mago & Salzberg, 2011). Sequences containing ambiguous ‘N’ were removed. Chimera sequences were detected and removed using UCHIME (Edgar et al., 2011). All sequences with 97% similarity were clustered using the USEARCH software to yield operational taxonomic units (OTUs). Low abundance OTUs (≤2 counts) were eliminated from the OTU table. Representative sequences for each OTU were assigned to taxonomic groups using UNITE database (Version 07.04.2014) (Abarenkov et al., 2010). In this study, all the sequences obtained were deposited in the SRA database short-read archive SRR5075731–SRR5075746.

### Statistical analysis

The Mothur software was used to calculate rarefaction and diversity indices of all the leaf samples based on resampling of OTUs generated by USEARCH (Schloss et al., 2009). Detrended correspondence analysis (DCA) and Venn diagram analysis were performed in subsequent analyses using the vegan package in R (v.3.2.5) (Oksanen et al., 2007). Community differences among the treatments were tested by using Nonparametric multi-response permutation procedures (MRPP, 999 permutations) based on Bray-Curtis distance methods in the R software package using the vegan package (v.3.2.5) (Anderson, 2001; Oksanen et al., 2007). The statistical significance of differences between groups (including Shannon index, inverse Simpson index and relative abundance of the taxonomic subgroups) was assessed by performing a one-way ANOVA followed by Tukey’s multiple comparison post hoc test when comparing several groups. The data are presented as the mean ± SE. Besides, a *P* value of <0.05 was considered to be statistically significant. The software IBM SPSS for Windows, version 22.0 was used to perform statistical analyses.

To determine whether the overall microbial communities present in the phyllosphere of pumpkin leaves with different disease levels were significantly different, nonparametric multi-response permutation procedures (MRPP) and **Adonis** were used based on Bray–Curtis distance methods in R package vegan (v.3.2.5) (Anderson, 2001; Oksanen et al., 2007).

## Results

### Fungi communities of the pumpkin phyllosphere

In total, 797,077 quality sequences were obtained for the four disease severity groups. The mean number of sequences per sample was 49,817, with a range of 39,028–62,150 sequences per sample. In total, 399 operational taxonomic units (OTUs) were detected using the UPARSE-OTU algorithm at the 97% identity cut-off (Tables S1 and S2). Rarefaction analysis and the Chao1 estimator indicated that the diversity in these leaf samples was within the same range (Fig. S1).

The four-way Venn diagrams in Fig. S2 show the distribution of the OTUs in the four disease severity groups. One hundred and fifty-five shared OTUs (38.8% of the total eukaryal OTUs) were found among four different groups. There were 10 (2.5%), 21 (5.2%), 14 (3.5%), and 5 OTUs (1.2%) of total eukaryal OTUs were only found in disease severity group L1, L2, L3 and L4, respectively (Fig. S2).

**Figure 1:**
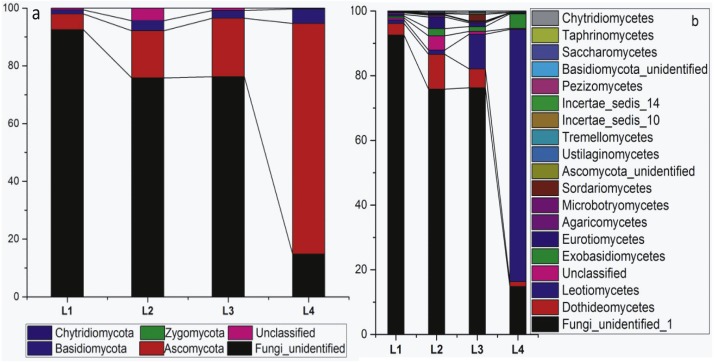
Relative abundance of fungal classification at the phylum and class level.

**Figure 2:**
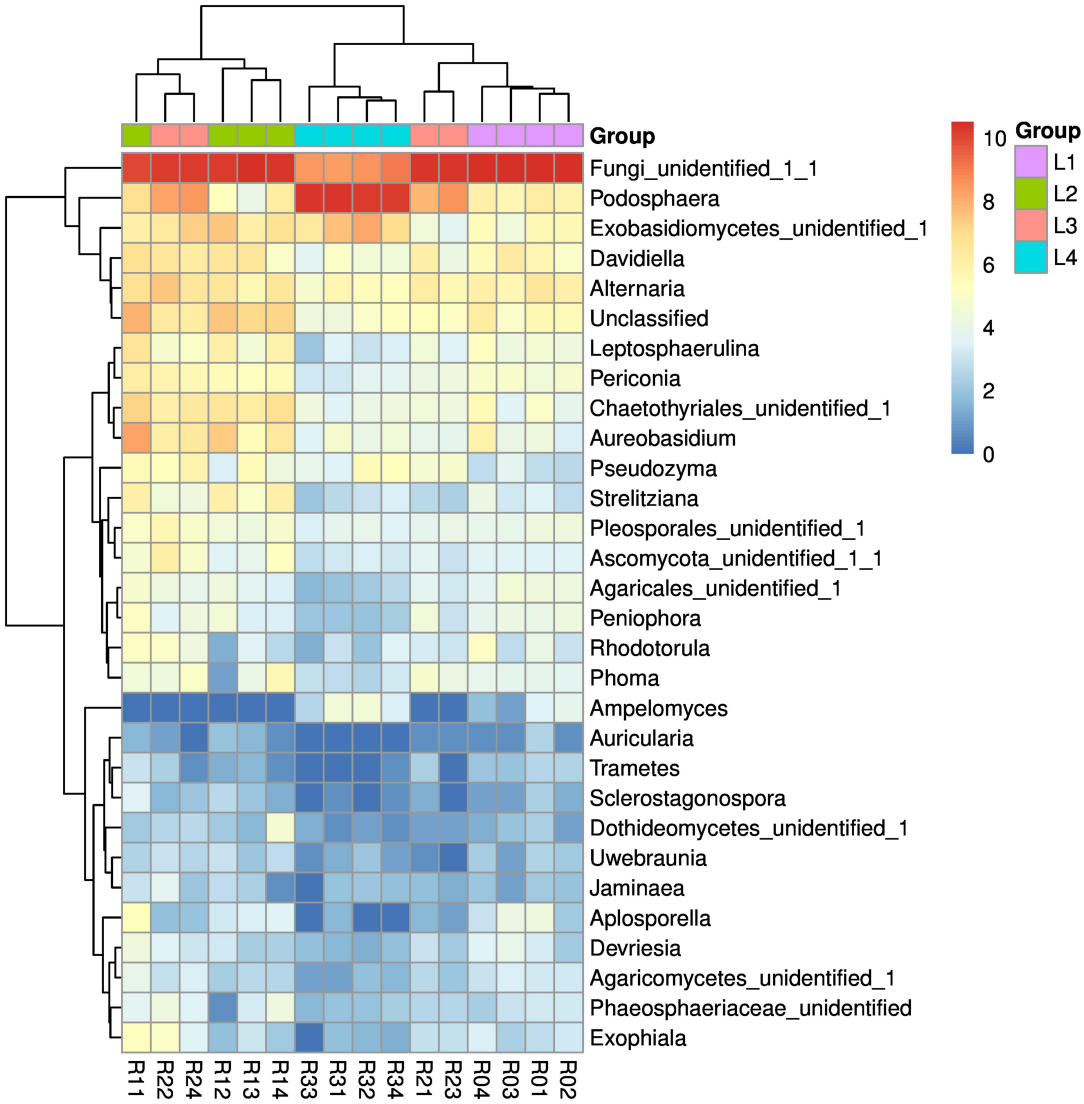
Heat map of the top 30 genera detected in all the samples. R01–R04, four replicate samples of the L1 level, R11–R14, four replicate samples of the L2 level, R21–R24, four replicate samples of the L3 level, R31–R34, four replicate samples of the L2 level. Different colors represent different relative abundances, red represents the high relative abundance, and green represents the low relative abundance. L1, L2, L3, and L4 are expressed in purple, green, pink, and blue, respectively.

Four fungal phyla, 15 classes and 36 orders were detected in the phyllosphere of the pumpkin samples (Table 1). The relative abundance of the main fungal phyllospheric populations at the taxonomic levels of Phyla and Class is shown in Figs. 1A and 1B, respectively. The abundance of Fungi_unidentified decreased while Ascomycota increased as disease severity increased with leaf. The heatmap of genus level indicated the most dominant genus was *Podosphaera* (Fig. 2), which showed different abundance among four disease severity groups.

**Table 1:** Number of detected phylotypes classified at different taxonomic levels.

Disease severity groups	Phylum	Class	Order	Family	Genus
No. of detected phylotypes	4	15	36	70	101
L1	3	14	35	63	86
L2	3	15	35	66	92
L3	3	14	31	68	87
L4	4	13	30	53	67

**Figure 3:**
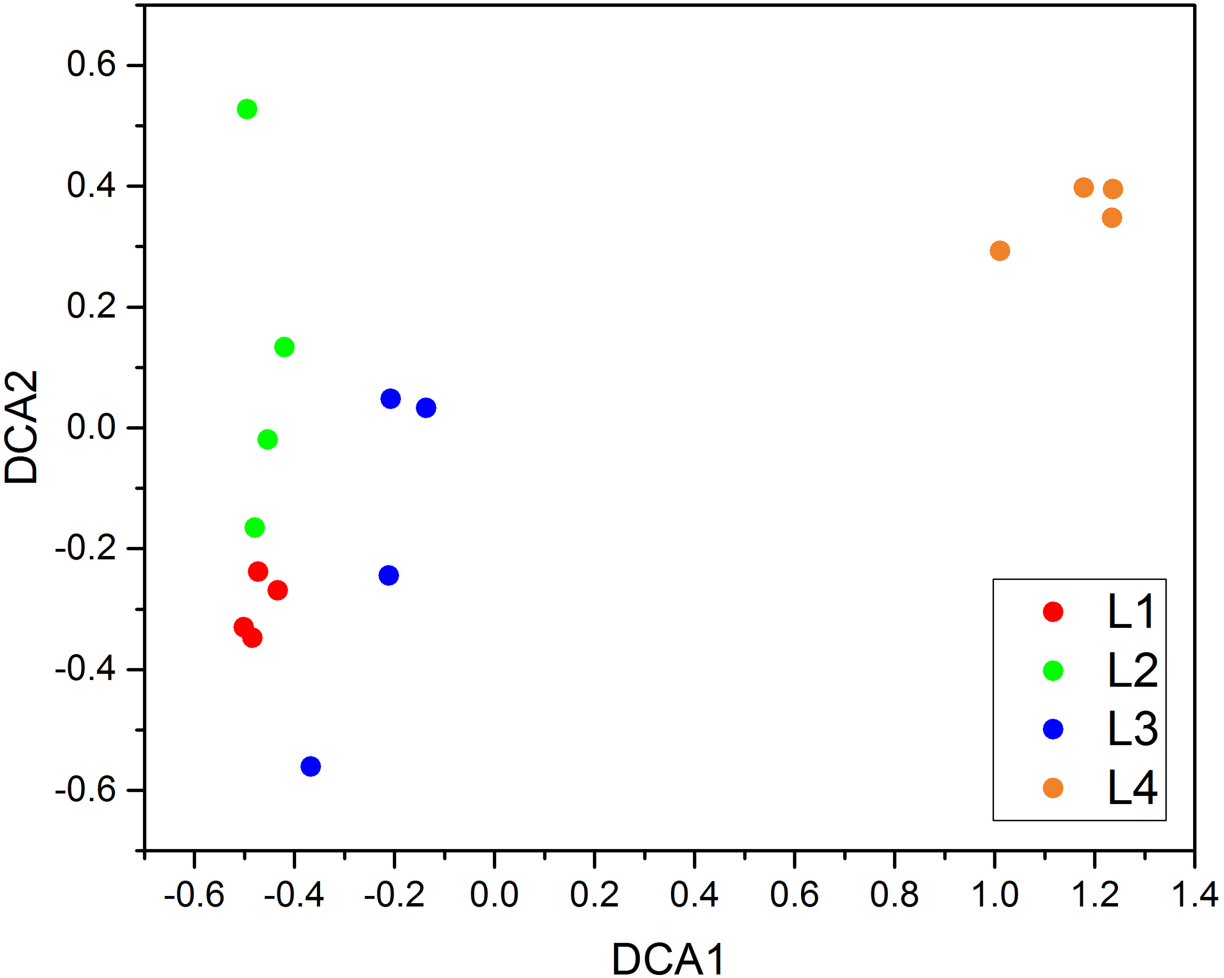
Detrended correspondence analysis (DCA). L1–L4 indicate the severity level of powdery mildew disease in each pumpkin leaf. *N* = 4.

Results of the MRPP analysis of fungal community composition showed an overall significant difference among four treatment levels based on the OTU table (*p* < 0.05) (Table S3). Adonis analysis also indicates that there was a significant difference between groups (*p* < 0.05) (Table S3). The detrended correspondence analysis (DCA) plot in Fig. 3 shows that the communities detected in leaves with different disease levels were clearly separated.

### Correlation between fungal communities and disease severity

We compared the fungal alpha diversity of the pumpkin leaves using the Shannon and Inverse Simpson diversity indices and OTU numbers (richness). The Shannon index ranged from 0.90 ± 0.09 to 1.87 ± 0.19, the Inverse Simpson index ranged from 1.61 ± 0.10 to 3.12 ± 0.53, and the richness ranged from 110.25 ± 6.85 to 217.00 ± 20.84 for the four disease severity groups. The results indicated that the fungal alpha diversity of the pumpkin leaves decreased significantly with increased disease severity from L2 to L4 (*p* < 0.05) (Table 2). However, alpha diversity in L2 leaves was higher than in L1 leaves.

**Figure 4:**
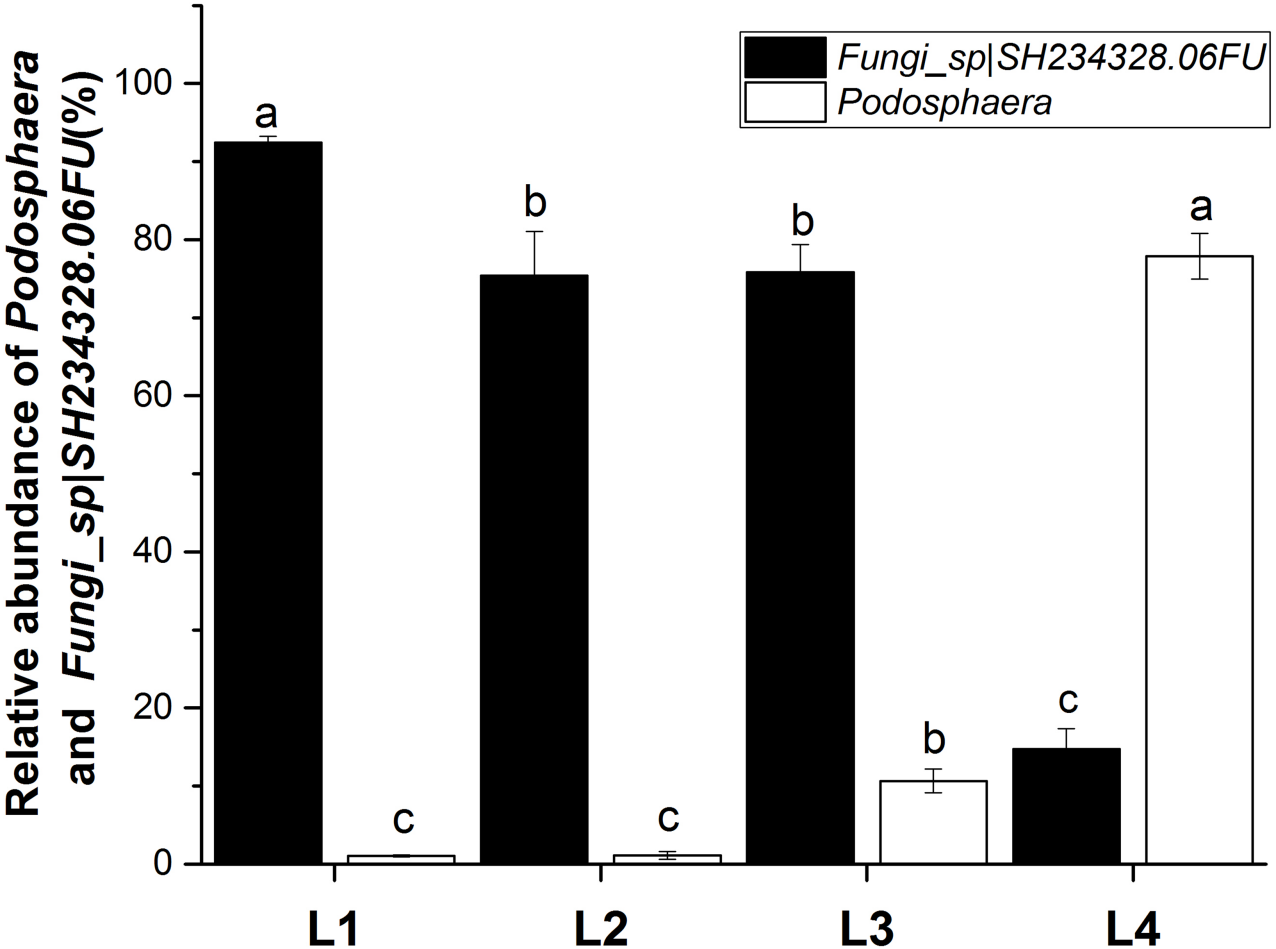
Relative abundance of *Podosphaera* and *Fungi_sp—SH234328.06FU* at different severity levels of powdery mildew disease (L1–L4).

The fungal communities were dominated by members of the Ascomycota and the most dominant genus was *Podosphaera* (Fig. 4). The abundance of Ascomycota and *Podosphaera* increased with increased disease severity. When the disease severity was greatest (L4), there was less fungal diversity but a greater number of OTUs showed a high level of abundance. 38.8% (155) were present in the phyllosphere of all the groups. The OTU_2, OTU_3, OTU_5, and OTU_9 were identified as Fungi_sp—SH234328.06FU (https://blast.ncbi.nlm.nih.gov/Blast.cgi, it is matched the sequence NCBI accession KF800560.1, as an uncultured eukaryote clone CMH469 18S ribosomal RNA gene, partial sequence, and the sequence similarity reached 98%) at the species level, and accounted for 92.42%, 75.41%, 75.85%, and 14.76% of the sequence reads detected in leaves at disease severity levels L1, L2, L3 and L4, respectively (*p* < 0.05) (Fig. 4). OTU_1 was identified as *Podosphaera_fusca*—SH194415.06FU, and accounted for 1.05%, 1.11%, 10.64%, and 77.9% of the sequence reads detected in leaves at disease severity levels L1, L2, L3 and L4, respectively (*p* < 0.05).

**Table 2:** Diversity indices of the communities on leaf surface showed different disease severity. The data are presented as the mean ± SE, a *P* value of <0.05 was considered to be statistically significant. The same letter indicates that there were no differences between groups, and different letters (a, b, c) indicate statistically significant differences.

Group	Richness	Shannon index	Inverse Simpson index	Chao1
L1	182.25 ± 4.53a	1.23 ± 0.03ab	2.03 ± 0.08ab	279.47 ± 10.47a
L2	217.00 ± 20.84a	1.87 ± 0.19c	3.12 ± 0.53c	283.08 ± 23.85a
L3	192.75 ± 27.19a	1.62 ± 0.16bc	2.64 ± 0.18bc	290.35 ± 22.21a
L4	110.25 ± 6.85b	0.90 ± 0.09a	1.61 ± 0.10a	181.91 ± 15.71b

## Disscusion

A number of studies have focused on the phyllosphere microorganisms in various plants, but the fungal community composition and diversity of pumpkin leaves infected with powdery mildew has not been reported. In our study, amplicon pyrosequencing of the ITS region of rDNA was used to detect the dynamics of fungal community response to different pumpkin powdery mildew disease severity. The dissimilarity among samples might be owing to the differences in the disease severity, and the pathogenic fungi which could select the related fungi colonize pumpkin leaf surface because of the symbiotic and competitive stresses among microbial species.

Microorganisms are the largest organisms on our planet and are an important component in the biogeochemical cycling of the earth. Microorganisms also play a crucial role in keeping leaves healthy (Baker et al., 2010) and in maintaining the balance of the ecosystem. Most microorganisms participate in the ecosystem cycle as decomposers. In addition, a variety of beneficial microorganisms colonized on the plant leaves can help to afford plant nutrition and defense against pathogens. Although there are many studies on the plant rhizosphere, there still lacks considerable attention and interest in the microbiology of leaf surfaces pathogens (Vorholt, 2012). Powdery mildew is a common fungal disease that can infect a wide range of plants, including cucurbits such as cucumbers, Luffa spp., melons and watermelons, leading to huge economic losses annually (Mcgrath & Shishkoff, 1999). Among the different species of fungi in the order Erysiphales causing powdery mildew, *Podosphaera xanthii* (a.k.a. *Sphaerotheca fuliginea*) is the most commonly reported cause (Mcgrath & Shishkoff, 1999). The development of high-throughout molecular techniques has helped us understand the microbial composition and structure in different environments and know how microbial diversity changes as the disease severity changes.

Our study has provided new insights into the impact of the plant pathogen *Podosphaera*, a serious pathogen that also causes pumpkin powdery mildew, on the microorganisms inhabiting the pumpkin phyllosphere. Previous studies have reported that there are usually more unique OTUs in the rhizosphere of healthy soil than in diseased soil (Rosenzweig et al., 2012). In the phyllosphere, there may be the same phenomenon as the soil. In our study, the greatest number of unique OTUs was found at disease severity level L2. Fungi_sp—SH234328.06FU was negatively correlated with disease severity (Fig. 4). There may be an antagonistic relationship between Fungi_sp—SH234328.06FU and *Podosphaera_fusca*—SH194415.06FU (*Podosphaera_xanthii*). We will investigate this relationship in a future study. The abundance of Ascomycota and *Podosphaera* was positively correlated with disease severity. As the pathogen of pumpkin powdery mildew, *Podosphaera* was the dominant genus in the heavy symptoms of mildew infection. DCA, MRPP and Adonis revealed significant differences in the composition and structure of the fungal assemblages observed in the four disease severity groups (Fig. 3, Table S3), suggesting that the composition and structure of the fungal assemblages altered as the disease severity increased.

The leaf fungal alpha diversity decreased significantly with increasing disease severity from L2 to L4 (Table 2). This result agrees with findings reported by Manching, Balintkurti & Stapleton (2014), who analyzed the relationship between southern leaf blight disease severity and maize leaf epiphytic bacterial species richness. It was found that lower species richness (alpha diversity) was correlated with an increase of southern leaf blight disease severity when disease pressure was higher. The decline in overall fungal diversity was enhanced after pathogen stimulation, which also agrees with the results reported by Erlacher et al. (2014). Interestingly, leaf fungal alpha diversity increased with increasing disease severity from L1 to L2, which suggests that the pathogen may have caused an increase in the fungal community richness at first and then a decrease when disease pressure was higher. It is well known that powdery mildew fungi are obligate biotrophs and will therefore compete for host nutrient reserves and suppress host defense responses. The growth and reproduction of other fungus could be inhibited when disease pressure was higher in the phyllosphere. This study further increases our understanding of the effect of powdery mildew disease on the microbial communities that inhabit the phyllosphere of pumpkin leaves. But there were some limitations in our study: first of all, a relative quantification of phyllosphere microbial populations by high-throughout sequencing unable to accurately determine the content of the species; second, a lack of greenhouse experiment control. In the subsequent experiments, we plan to study phyllosphere microbial communities’ response to different disease severity of pumpkin powdery mildew in the field and greenhouse by artificial inoculation and qPCR quantitative detection.

## Conclusions

In our current study, we demonstrated that the plant pathogen *Podosphaera_fusca* can affect the phyllosphere fungal communities of pumpkin. The pathogen caused an increase in the fungal community richness at first and then a decrease when disease pressure was higher. The decline in overall fungal diversity was enhanced after pathogen stimulation. The abundance of an unidentified genus as Fungi_sp—SH234328.06FU was inversely proportional to pathogen community of *Podosphaera*. It could give new perspectives on the changes in the leaf microbiome at different pumpkin powdery mildew disease severities.

##  Supplemental Information

10.7717/peerj.4559/supp-1 Figure S1 Rarefaction curves for the operational taxonomic units (OTUs) R01–R04: four replicate samples of the L1 level; R11–R14: four replicate samples of the L2 level; R21–R24: four replicate samples of the L3 level; R31–R34: four replicate samples of the L2 level. L1, L2, L3, and L4 are expressed in red, green, blue and purple, respectively.

10.7717/peerj.4559/supp-2 Figure S2 Venn diagram showing unique and shared OTUs detected in the phyllosphere of the four disease severity groups (L1, L2, L3 and L4)

10.7717/peerj.4559/supp-3 Table S1 The unique and shared OTUs and their taxonomic annotation in the four different samples

10.7717/peerj.4559/supp-4 Table S2 Taxonomic information of each OTU ID

10.7717/peerj.4559/supp-5 Table S3 Statistical analysis of the microbial community composition and structure detected at different disease severity levels

## Additional Information and Declarations

### Data Availability

The following information was supplied regarding data availability: The sequences obtained were deposited in the SRA database short-read archive SRR5075731–SRR5075746.
