# Supplementary figures and images for: Pumpkin powdery mildew disease severity influences the fungal diversity of the phyllosphere
Source: PeerJ. 2018 Apr 2;6:e4559. doi: 10.7717/peerj.4559 (PMC5885987; doi:10.7717/peerj.4559)

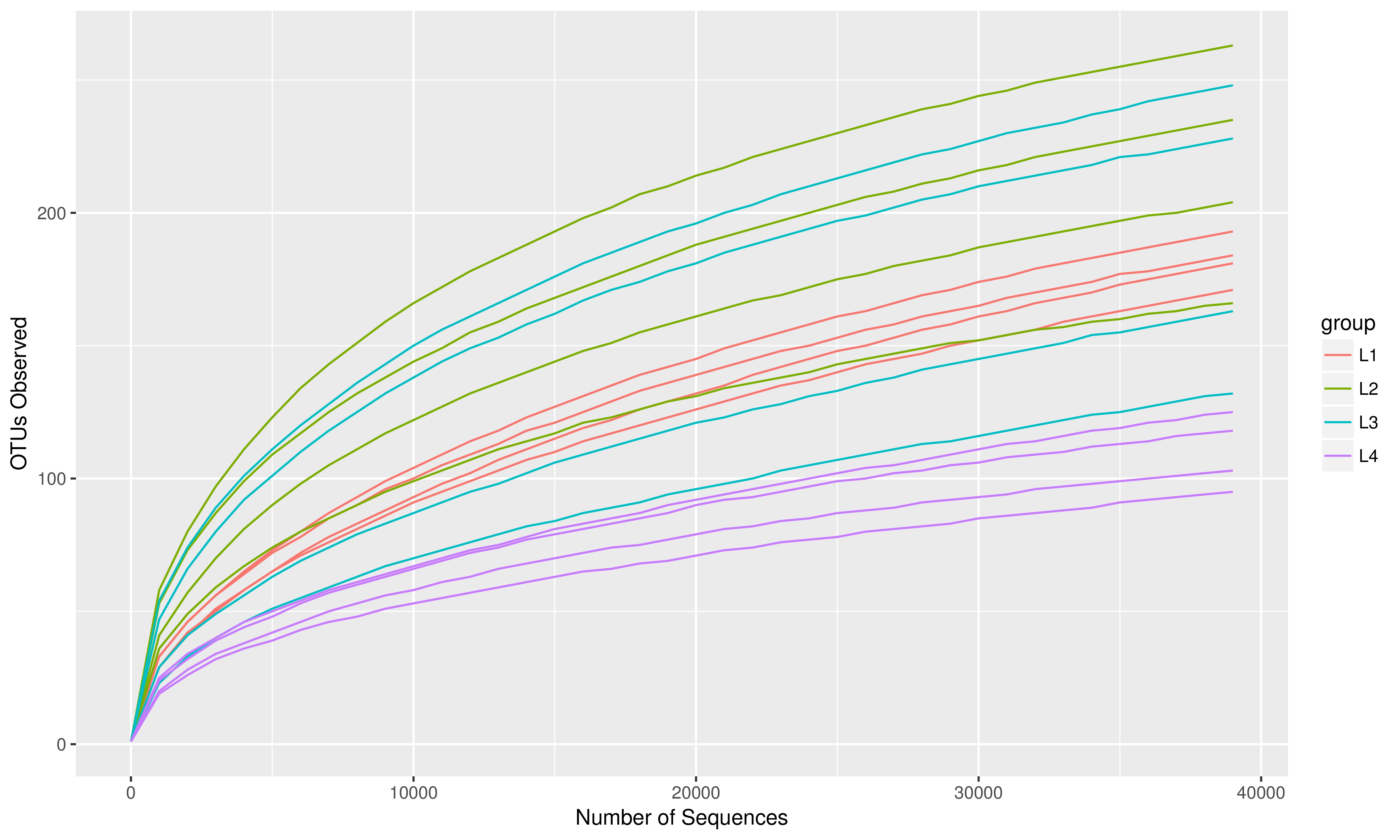

Supplement: Figure S1 — R01–R04: four replicate samples of the L1 level; R11–R14: four replicate samples of the L2 level; R21–R24: four replicate samples of the L3 level; R31–R34: four replicate samples of the L2 level. L1, L2, L3, and L4 are expressed in red, green, blue and purple, respectively. [file peerj-06-4559-s001.jpeg]
