# Supplementary material for: Pumpkin powdery mildew disease severity influences the fungal diversity of the phyllosphere
Source: PeerJ. 2018 Apr 2;6:e4559. doi: 10.7717/peerj.4559 (PMC5885987; doi:10.7717/peerj.4559)
Supplement: Table S1 [file peerj-06-4559-s003.docx]

**Table S1 The unique and shared OTUs and their taxonomic** [**annotation**](https://bmcbioinformatics.biomedcentral.com/articles/10.1186/1471-2105-12-182) **in the four different samples.**

|  | **Genus** | **L1** | **L2** | **L3** | **L4** |
| --- | --- | --- | --- | --- | --- |
| OTU1 | Podosphaera_fusca\|SH194415.06FU | + | + | + | + |
| OTU10 | Davidiella_tassiana\|SH196750.06FU | + | + | + | + |
| OTU100 | Unclassified_Ascomycota | + | + | + | + |
| OTU101 | Leucosporidiella_fragaria\|SH212317.06FU | + | + | + | + |
| OTU113 | Agaricomycetes_sp\|SH234602.06FU | + | + | + | + |
| OTU12 | Leptosphaerulina_chartarum\|SH227423.06FU | + | + | + | + |
| OTU13 | Pseudozyma_sp_Vega417\|SH241684.06FU | + | + | + | + |
| OTU14 | Strelitziana_mali\|SH211991.06FU | + | + | + | + |
| OTU143 | Unclassified_Fungi | + | + | + | + |
| OTU16 | Exobasidiomycetes_sp\|SH230059.06FU | + | + | + | + |
| OTU2 | Fungi_sp\|SH234328.06FU | + | + | + | + |
| OTU20 | Rhodotorula_sp_YM24636\|SH227561.06FU | + | + | + | + |
| OTU15 | Exobasidiomycetes_sp\|SH199847.06FU | + | + | + | + |
| OTU17 | Periconia_sp\|SH232015.06FU | + | + | + | + |
| OTU18 | Chaetothyriales_sp\|SH201524.06FU | + | + | + | + |
| OTU21 | Agaricales_sp\|SH239393.06FU | + | + | + | + |
| OTU22 | Helotiales_sp\|SH191096.06FU | + | + | + | + |
| OTU23 | Pleosporales_sp\|SH206981.06FU | + | + | + | + |
| OTU26 | Pestalotiopsis_rhododendri\|SH210426.06FU | + | + | + | + |
| OTU27 | Fungi_sp\|SH201526.06FU | + | + | + | + |
| OTU273 | Ascomycota_sp\|SH202146.06FU | + | + | + | + |
| OTU281 | Pleosporales_sp\|SH206981.06FU | + | + | + | + |
| OTU3 | Fungi_sp\|SH234328.06FU | + | + | + | + |
| OTU30 | Unclassified_Devriesia | + | + | + | + |
| OTU24 | Chaetothyriales_sp\|SH232791.06FU | + | + | + | + |
| OTU35 | Phaeosphaeriaceae_sp\|SH213437.06FU | + | + | + | + |
| OTU36 | Unclassified_Chaetothyriales | + | + | + | + |
| OTU37 | Penicillium_sp\|SH193634.06FU | + | + | + | + |
| OTU38 | Neodeightonia_palmicola\|SH236115.06FU | + | + | + | + |
| OTU164 | Unclassified_Pleosporales | + | + | + | + |
| OTU19 | Phoma_microchlamydospora\|SH197540.06FU | + | + | + | + |
| OTU32 | Chaetothyriales_sp\|SH201524.06FU | + | + | + | + |
| OTU33 | Peniophora_sp_JY_035\|SH234595.06FU | + | + | + | + |
| OTU102 | Psathyrella_candolleana\|SH199666.06FU | + | + | + | - |
| OTU103 | Phaeosphaeria_fuckelii\|SH240851.06FU | + | + | + | - |
| OTU4 | Exobasidiomycetes_sp\|SH230059.06FU | + | + | + | + |
| OTU40 | Mycosphaerella_gregaria\|SH235741.06FU | + | + | + | + |
| OTU41 | Exophiala_brunnea\|SH230205.06FU | + | + | + | + |
| OTU42 | Jaminaea_angkoriensis\|SH233453.06FU | + | + | + | + |
| OTU44 | Coniothyrium_sp_JK27\|SH233607.06FU | + | + | + | + |
| OTU45 | Fungi_sp\|SH201526.06FU | + | + | + | + |
| OTU46 | Ascomycota_sp\|SH194735.06FU | + | + | + | + |
| OTU47 | Strelitziana_albiziae\|SH211993.06FU | + | + | + | + |
| OTU5 | Fungi_sp\|SH234328.06FU | + | + | + | + |
| OTU50 | Agaricomycetes_sp\|SH205543.06FU | + | + | + | + |
| OTU52 | Chaetothyriales_sp\|SH219207.06FU | + | + | + | + |
| OTU54 | Unclassified_Ascomycota | + | + | + | + |
| OTU58 | Fungi_sp\|SH212314.06FU | + | + | + | + |
| OTU60 | Toxicocladosporium_strelitziae\|SH196751.06FU | + | + | + | + |
| OTU61 | Ascomycota_sp\|SH191097.06FU | + | + | + | + |
| OTU63 | Unclassified_Myrothecium | + | + | + | + |
| OTU6 | Alternaria_eichhorniae\|SH224789.06FU | + | + | + | + |
| OTU7 | Aureobasidium_sp\|SH206630.06FU | + | + | + | + |
| OTU70 | Strelitziana_albiziae\|SH211993.06FU | + | + | + | + |
| OTU72 | Leptospora_rubella\|SH213116.06FU | + | + | + | + |
| OTU73 | Uwebraunia_musae\|SH197937.06FU | + | + | + | + |
| OTU31 | Phoma_aff_macrostoma_BBA_72146\|SH202148.06FU | + | + | + | + |
| OTU65 | Unclassified_Chaetothyriales | + | + | + | + |
| OTU67 | Chaetothyriales_sp\|SH214498.06FU | + | + | + | + |
| OTU89 | Ascomycota_sp\|SH226078.06FU | + | + | + | + |
| OTU9 | Fungi_sp\|SH234328.06FU | + | + | + | + |
| OTU96 | Diaporthe_caulivora\|SH194728.06FU | + | + | + | + |
| OTU75 | Stagonosporopsis_tanaceti\|SH202147.06FU | + | + | + | + |
| OTU8 | Unclassified_Fungi | + | + | + | + |
| OTU105 | Chaetothyriales_sp\|SH201524.06FU | - | + | - | - |
| OTU107 | Unclassified_Ascomycota | + | + | + | - |
| OTU109 | Unclassified_Chaetothyriales | - | + | - | - |
| OTU11 | Cordycipitaceae_sp\|SH235767.06FU | + | + | + | - |
| OTU112 | Unclassified_Rigidoporus | + | + | - | + |
| OTU115 | Microbotryozyma_collariae\|SH238724.06FU | + | + | + | - |
| OTU116 | Dothideomycetes_sp\|SH225319.06FU | - | + | - | - |
| OTU120 | Coniothyrium_sidae\|SH196237.06FU | + | + | + | - |
| OTU121 | Unclassified_Ascomycota | + | + | - | - |
| OTU123 | Sarocladium_strictum\|SH191575.06FU | + | - | - | - |
| OTU125 | Diatrypaceae_sp\|SH227086.06FU | - | - | + | - |
| OTU128 | Fungi_sp\|SH201526.06FU | - | + | - | - |
| OTU129 | Sordariomycetes_sp\|SH212488.06FU | + | + | + | - |
| OTU130 | Pyrenochaeta_lycopersici\|SH240064.06FU | - | - | - | + |
| OTU131 | Toxicocladosporium_strelitziae\|SH196751.06FU | - | + | - | - |
| OTU135 | Hannaella_sp_CMON52\|SH197181.06FU | + | + | + | - |
| OTU136 | Unclassified_Ascomycota | - | + | - | - |
| OTU137 | Psathyrella_candolleana\|SH199666.06FU | + | + | + | - |
| OTU138 | Unclassified_Mycosphaerellaceae | + | + | + | - |
| OTU139 | Rhodotorula_acheniorum\|SH191070.06FU | - | - | + | - |
| OTU140 | Unclassified_Fungi | + | - | - | - |
| OTU141 | Leptosphaerulina_chartarum\|SH227423.06FU | - | + | - | - |
| OTU142 | Chaetothyriales_sp\|SH201524.06FU | - | + | - | - |
| OTU144 | Whalleya_microplaca\|SH227044.06FU | - | - | + | - |
| OTU145 | Unclassified_Chaetothyriales | + | + | + | - |
| OTU147 | Phoma_brasiliensis\|SH202145.06FU | + | + | + | - |
| OTU148 | Leptosphaeriaceae_sp\|SH206994.06FU | + | + | + | - |
| OTU149 | Agaricomycetes_sp\|SH214757.06FU | + | - | - | - |
| OTU150 | Phoma_brasiliensis\|SH202145.06FU | + | + | + | - |
| OTU151 | Unclassified_Agaricales | + | - | - | - |
| OTU152 | Diaporthe_phaseolorum\|SH194772.06FU | - | - | + | - |
| OTU153 | Unclassified_Mycosphaerellaceae | - | + | - | - |
| OTU155 | Pezizomycetes_sp\|SH212017.06FU | + | - | - | - |
| OTU159 | Schizopora_ovispora\|SH204974.06FU | + | - | - | - |
| OTU160 | Ophiognomonia_sogonovii\|SH226094.06FU | - | + | - | - |
| OTU162 | Ascomycota_sp\|SH224125.06FU | - | + | - | - |
| OTU163 | Unclassified_Chaetothyriales | - | + | - | - |
| OTU165 | Unclassified_Pleosporales | - | - | + | - |
| OTU167 | Unclassified_Pleosporales | + | - | - | - |
| OTU169 | Toxicocladosporium_strelitziae\|SH196751.06FU | - | + | - | - |
| OTU170 | Devriesia_fraseriae\|SH189975.06FU | + | + | + | - |
| OTU172 | Leptospora_rubella\|SH213116.06FU | - | - | + | - |
| OTU181 | Toxicocladosporium_strelitziae\|SH196751.06FU | + | - | - | - |
| OTU182 | Sporidiobolales_sp\|SH196708.06FU | + | + | + | - |
| OTU183 | Unclassified_Ascomycota | - | + | - | - |
| OTU187 | Unclassified_Xylodon | + | - | - | - |
| OTU190 | Mycosphaerella_gregaria\|SH235741.06FU | - | + | - | - |
| OTU191 | Pleosporales_sp\|SH196178.06FU | - | - | + | - |
| OTU196 | Pleosporales_sp\|SH231086.06FU | - | - | + | - |
| OTU199 | Sordariomycetes_sp\|SH219629.06FU | - | + | - | - |
| OTU201 | Unclassified_Mycosphaerellaceae | - | + | - | - |
| OTU207 | Pseudocercospora_dovyalidis\|SH212656.06FU | - | + | - | - |
| OTU211 | Unclassified_Sporobolomyces | + | - | - | - |
| OTU212 | Unclassified_ | + | + | + | - |
| OTU216 | Russulales_sp\|SH234592.06FU | - | - | + | - |
| OTU226 | Ascomycota_sp\|SH189849.06FU | - | - | + | - |
| OTU237 | Phoma_brasiliensis\|SH202145.06FU | - | + | - | - |
| OTU238 | Mycosphaerellaceae_sp\|SH212680.06FU | - | + | - | - |
| OTU239 | Coprinopsis_urticicola\|SH229042.06FU | - | + | - | - |
| OTU243 | Chaetothyriaceae_sp\|SH210957.06FU | - | - | + | - |
| OTU245 | Exophiala_brunnea\|SH230205.06FU | - | - | + | - |
| OTU25 | Unclassified_Aplosporella | + | + | + | - |
| OTU256 | Psathyrellaceae_sp\|SH239392.06FU | - | - | + | - |
| OTU269 | Unclassified_Incertae_sedis_16 | - | + | + | + |
| OTU278 | Ascomycota_sp\|SH229418.06FU | - | + | - | - |
| OTU288 | Gibberella_zeae\|SH217300.06FU | - | + | - | - |
| OTU29 | Ascomycota_sp\|SH224125.06FU | - | - | + | - |
| OTU299 | Pleosporales_sp\|SH209063.06FU | - | + | - | - |
| OTU309 | Unclassified_Helotiales | - | - | + | - |
| OTU312 | Sordariomycetes_sp\|SH219632.06FU | - | + | - | - |
| OTU315 | Helotiales_sp\|SH191096.06FU | - | + | - | - |
| OTU330 | Sordariomycetes_sp\|SH212492.06FU | - | - | + | - |
| OTU332 | Unclassified_Ascomycota | - | + | - | - |
| OTU338 | Strelitziana_africana\|SH211992.06FU | - | + | - | - |
| OTU34 | Unclassified_Hypocreales | + | + | + | - |
| OTU388 | Ascomycota_sp\|SH224125.06FU | - | + | - | - |
| OTU39 | Fungi_sp\|SH234328.06FU | + | + | + | - |
| OTU396 | Cryptococcus_aff_taibaiensis_IMUFRJ_51982\|SH198061.06FU | + | - | - | - |
| OTU48 | Exophiala_brunnea\|SH230205.06FU | + | + | + | - |
| OTU49 | Trametes_hirsuta\|SH236971.06FU | + | + | + | - |
| OTU53 | Arthrinium_malaysianum\|SH233379.06FU | + | + | + | - |
| OTU55 | Ustilaginaceae_sp\|SH241685.06FU | - | + | + | + |
| OTU56 | Phaeosphaeriaceae_sp\|SH206983.06FU | + | + | + | - |
| OTU57 | Pyricularia_sp\|SH230953.06FU | + | + | - | + |
| OTU59 | Ascomycota_sp\|SH224125.06FU | + | + | + | - |
| OTU64 | Cyphellophora_pluriseptata\|SH214503.06FU | + | + | + | - |
| OTU66 | Choanephora_cucurbitarum\|SH218423.06FU | + | + | + | - |
| OTU68 | Cyphellophora_suttonii\|SH232792.06FU | - | + | + | + |
| OTU71 | Unclassified_Agaricomycetes | + | + | + | - |
| OTU74 | Leptospora_rubella\|SH213116.06FU | + | + | + | - |
| OTU76 | Ascomycota_sp\|SH211264.06FU | - | + | - | - |
| OTU77 | Exobasidiomycetes_sp\|SH199847.06FU | + | + | - | + |
| OTU78 | Ascomycota_sp\|SH224125.06FU | + | + | - | + |
| OTU79 | Marasmius_maximus\|SH219756.06FU | + | + | + | - |
| OTU80 | Unclassified_Fungi | + | + | + | - |
| OTU82 | Sclerostagonospora_sp_CBS_118152\|SH224099.06FU | + | + | + | - |
| OTU83 | Agaricales_sp\|SH239391.06FU | + | + | + | - |
| OTU84 | Unclassified_Cyphellophora | + | + | + | - |
| OTU85 | Ustilago_longissima_var._macrospora\|SH237194.06FU | - | - | + | - |
| OTU87 | Auricularia_sp_12204\|SH212879.06FU | + | + | + | - |
| OTU90 | Mucor_circinelloides_f_lusitanicus\|SH226012.06FU | + | + | + | - |
| OTU91 | Unclassified_Peniophoraceae | + | - | - | - |
| OTU92 | Ascomycota_sp\|SH240077.06FU | + | + | + | - |
| OTU94 | Fungi_sp\|SH213113.06FU | + | + | + | - |
| OTU95 | Strelitziana_albiziae\|SH211993.06FU | + | + | + | - |
| OTU97 | Fungi_sp\|SH192549.06FU | - | + | + | + |
| OTU98 | Dissoconium_proteae\|SH197939.06FU | + | + | - | + |
| OTU99 | Cyphellophora_suttonii\|SH232792.06FU | - | + | - | - |

+: detected, -: not detected.
