# Supplementary material for: Pumpkin powdery mildew disease severity influences the fungal diversity of the phyllosphere
Source: PeerJ. 2018 Apr 2;6:e4559. doi: 10.7717/peerj.4559 (PMC5885987; doi:10.7717/peerj.4559)
Supplement: Table S3 [file peerj-06-4559-s005.docx]

**Table S3.** Statistical analysis of the microbial community composition and structure detected at different disease severity levels

| **Disease severity levels** | **MRPP** | | **Adonis** | |
| --- | --- | --- | --- | --- |
|  | ***P*** | **Delta** | ***P*** | **R^2^** |
| L1–L2 | 0.03 | 0.145 | 0.001 | 0.392 |
| L1–L3 | 0.037 | 0.113 | 0.001 | 0.539 |
| L1–L4 | 0.029 | 0.089 | 0.001 | 0.98 |
| L2–L3 | 0.03 | 0.178 | 0.021 | 0.346 |
| L2–L4 | 0.038 | 0.153 | 0.014 | 0.936 |
| L3–L4 | 0.034 | 0.121 | 0.014 | 0.953 |

*P* value of <0.05 was considered to be statistically significant. MRPP, multiple-response permutation procedure.
